# How to Improve Meniscal Repair through Biological Augmentation: A Narrative Review

**DOI:** 10.3390/jcm13164688

**Published:** 2024-08-09

**Authors:** Pierangelo Za, Luca Ambrosio, Sebastiano Vasta, Fabrizio Russo, Giuseppe Francesco Papalia, Gianluca Vadalà, Rocco Papalia

**Affiliations:** 1 Operative Research Unit of Orthopaedic and Trauma Surgery, Fondazione Policlinico Universitario Campus Bio-Medico, 00128 Rome, Italy; p.za@unicampus.it (P.Z.); s.vasta@policlinicocampus.it (S.V.); fabrizio.russo@policlinicocampus.it (F.R.); g.papalia@policlinicocampus.it (G.F.P.); g.vadala@policlinicocampus.it (G.V.); r.papalia@policlinicocampus.it (R.P.); 2 Research Unit of Orthopaedic and Trauma Surgery, Department of Medicine and Surgery, Università Campus Bio-Medico di Roma, 00128 Rome, Italy

**Keywords:** meniscus, meniscal repair, knee, arthroscopy, sports medicine, meniscectomy, platelet-rich plasma, microfracture

## Abstract

Since the role of the menisci in knee stability, proprioception, and homeostasis has been well established, significant efforts have been made to repair meniscal tears, resulting in excellent clinical outcomes and a reduction in the progression of knee osteoarthritis (OA). However, varying failure rates have been reported, raising questions regarding the healing potential in cases of complex injuries, poorly vascularized and degenerated areas, and generally in the presence of unfavorable biological characteristics. Therefore, over the last few decades, different strategies have been described to increase the chances of meniscal healing. Biological augmentation of meniscal repair through various techniques represents a safe and effective strategy with proven clinical benefits. This approach could reduce the failure rate and expand the indications for meniscal repair. In the present study, we thoroughly reviewed the available evidence on meniscal repair surgery and summarized the main techniques that can be employed to enhance the biological healing potential of a meniscal lesion. Our aim was to provide an overview of the state of the art on meniscal repair and suggest the best techniques to reduce their failure rate.

## 1. Introduction

Meniscectomy was once a widely performed procedure [1,2,3,4] and considered the gold standard for treating symptomatic meniscal tears, offering good short-term clinical results, pain resolution, and functional improvement [5]. However, recent studies have questioned the effectiveness of meniscectomy [2,6,7,8,9]. Good clinical outcomes depend on meticulous surgical indications, suggesting that partial meniscectomy should be reserved for patients with mechanical symptoms and unstable meniscal lesions on magnetic resonance imaging (MRI), or those who did not respond to conservative treatment [10]. Additionally, arthroscopic meniscectomy has shown no superiority over sham surgery in cases of degenerative meniscopathy [9], and unsatisfactory results are to be expected if meniscectomy is performed without mechanical symptoms [11]. It is also essential to differentiate between medial and lateral meniscectomy. While the former has been linked to better functional outcomes [12], the latter has been associated with an increased risk of chondrolysis [13,14] and less favorable clinical results [15]. Furthermore, factors such as the integrity of the meniscal roots, lower limb alignment, presence of chondral lesions, concomitant instability, and body mass index (BMI) need to be considered when contemplating a meniscectomy [12,16,17,18,19].

For years, meniscectomy was unquestionably performed worldwide until long-term studies demonstrated a direct correlation with the progression of knee osteoarthritis (OA) [20,21,22,23,24]. This called into question the long-term effectiveness of meniscectomy and paved the way for the introduction of meniscal repair techniques. Meniscal repair has become a crucial consideration not only to improve clinical outcomes, but also for ethical and economic reasons. Indeed, the increasing incidence of OA leads to a higher number of joint replacement surgeries, thus placing a substantial financial burden on the healthcare systems [25]. Hence, the prevention of OA is essential from both cost and benefit perspectives.

We conducted a narrative review of the literature to highlight current advancements in meniscal repair and outline the primary techniques commonly employed to enhance the likelihood of biological healing in meniscal repair.

## 2. Materials and Methods

### 2.1. Literature Search Strategy

We conducted a comprehensive state-of-the art literature search using databases such as PubMed, Scopus, and Google Scholar. The search covered articles published up to February 2024. Key terms included “meniscal repair” OR “meniscus repair” AND “biological augmentation”.

### 2.2. Inclusion and Exclusion Criteria

Eligible studies were peer-reviewed articles written in English that involved human subjects and specifically reported on the outcomes and techniques of meniscal repair and its biological augmentation. Exclusion criteria included case reports, non-English publications, studies with insufficient outcome data, and animal studies.

### 2.3. Data Extraction

Data extraction was performed by two independent reviewers (P.Z, L.A.). Demographic data, clinical and radiological outcomes, reoperation, and failure rates were extrapolated and collected in tables and synthesized narratively.

## 3. Why Repair?

Both the medial and the lateral menisci are important stabilizers and proprioceptors of the knee [26,27]. Meniscectomy alters these properties and results in elevated contact pressure in the femorotibial compartment, directly related to the extent of meniscal tissue removal [28,29,30,31,32]. It also disrupts knee kinematics and stability [21,33,34], promoting cartilage wear and OA progression [35,36]. The principle behind meniscal tissue repair is the potential for healing particularly due to the rich vascularization in the red-red zone and partly in the red-white zone of the meniscus.

Various methods for meniscal repair have been developed, including all-inside, out–in, and in–out techniques [37,38]. The selection of the best approach among these depends on the nature and location of the meniscal injury, along with the surgeon’s preference. When assessing the outcomes of meniscal repair, it is crucial to consider the specifics of the technique and devices used, the nature of the lesion, its location, and the characteristics of the population examined. The failure rate may also vary depending on the definition of failure adopted by different authors. Consequently, meniscal repair failure rates are highly variable in the literature [15,39,40,41,42,43]. Duethman et al. [15] reported a non-statistically significant difference in failure and reoperation rates between meniscal repair and meniscectomy groups. Conversely, Paxton et al. [43] reported a higher failure rate for meniscal repair. On the other hand, other authors [44,45,46] reported significant improvements in patient outcomes following meniscal repair compared to meniscectomy. Table 1 depicts the main studies comparing the results of meniscectomy versus meniscal repair [15,42,43,47,48,49,50,51,52].

In the authors’ opinion, the current failure rates of meniscal repair are unsatisfactory and need to be improved. However, surgeons should not be discouraged from performing meniscal repairs. When a repair fails, it is often possible to perform a new repair or a meniscectomy, which is usually less invasive than what would have been required initially. This approach allows for excellent long-term clinical outcomes even after the failure of a meniscal repair requiring revision [53]. Hagmeijer et al. [53] reported zero cases of failure after revising a meniscal repair at a mean follow-up of 17.6 years. Similarly, D’Ambrosi et al. [50] reported a return to sport rate of over 80% in a population of professional athletes. The causes of failure after meniscectomy include persistent pain, instability, and chondrolysis, complications that are challenging to manage and may necessitate more aggressive interventions such as a new meniscectomy, osteotomy, or osteochondral or meniscal allograft implantation. In contrast, the most common causes of failure after repair are nonspecific pain and suture failure, which can be more easily managed with a new repair or partial meniscectomy [50]. However, the failure rate of repairs remains high, particularly within one year after surgery [42,53]. Complex and bucket handle tears have the highest failure rates at 80% and 47%, respectively, while isolated meniscal lesions have a failure rate of 18.2% [53]. Isolated repair has a higher failure rate than meniscal repair with concomitant anterior cruciate ligament reconstruction (ACLr) [43,54,55,56,57,58]. A recent meta-analysis examining meniscal repair outcomes in 3829 patients with a minimum follow-up of two years documented an overall failure rate of 14.8%, which dropped to 8.5% in the presence of a concomitant ACLr [59]. Furthermore, medial meniscus repair tends to fail more often than lateral meniscus repair, while partial lateral meniscectomy has a higher reintervention rate compared to partial medial meniscectomy [43,50]. Recent data report failure rates of 6.1% and 10.8% for repairs of the lateral and medial meniscus, respectively. Repairs in adults do not have lower failure rates compared to younger people, indicating that advanced age does not adversely affect the failure rate. Therefore, it is not contraindicated to perform meniscal repair in patients over the age of 40 [39,54,56,60,61,62]. Although there is no consensus, Wouters et al. [63] recently reported that repairing a meniscal injury within three weeks of injury reduces the failure rate. Most failures occur in the first year due to impaired healing or reinjury [40,53]. The implementation of an early range of motion and immediate postoperative weightbearing does not appear to negatively impact the likelihood of clinical success following meniscal repair [64]. Key data on meniscal repair failure rates are summarized in Table 2 and Table 3.

However, biological healing time should be respected before returning to sport, which should not be early in the case of meniscal repair, especially in the young population. The current challenge is to lower the failure rate of a meniscal repair. Several techniques have been described to promote meniscal healing after repair.

## 4. Meniscal and Biological Augmentation

**Figure 1:**
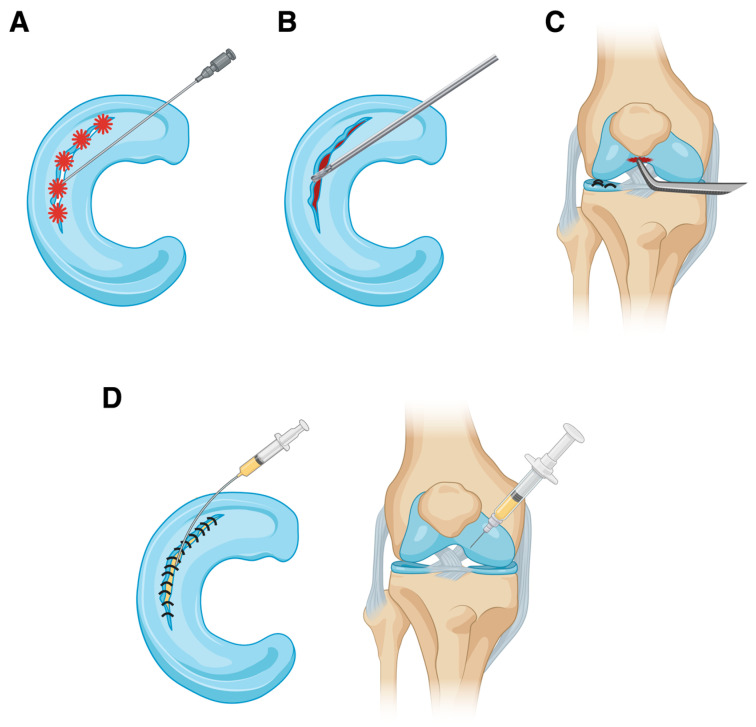
The main techniques to perform biological augmentation of repaired meniscal tears include trephination (**A**), abrasion (**B**), microfracture (**C**), and the administration of platelet-rich plasma, fibrin clots, or platelet-rich fibrin clots (**D**). These can be delivered directly into the repaired tear or injected into the knee joint after surgery. Created with BioRender.com.

Over time, various techniques have been developed to enhance the chances of successful meniscal healing. The most common augmentation techniques for meniscal repair are shown Figure 1 and summarized in Table 4.

### 4.1. Trephination

Trephination is a straightforward technique wherein a spinal needle is arthroscopically used to drill holes in the red-red zone, creating multiple channels to divert blood flow from a vascularized area to a less vascularized area. This allows growth factors and cells to reach the meniscal lesion, stimulating fibrous scar formation and its remodeling into fibrocartilaginous tissue [69,70]. Experimental studies on animals [70,71,72] have shown that trephination increases vascularization and improves tissue healing. In humans, this translates into enhanced clinical outcomes when trephinations are added to meniscal repair [73,74]. However, the effectiveness of trephinations as an isolated procedure is still debated [72].

### 4.2. Abrasion

Abrasion of the margins of the meniscal lesion and the parameniscal synovium can be performed using a shaver or rasp. This easy and fast mechanical stimulation elicits a damage response with the release of growth factors and cytokines that trigger a process of revascularization and healing [75,76]. Rasping the parameniscal synovium and abrading the tear edges seem to yield superior results compared to fibrin clots in murine [77] and rabbit models [78]. Excellent healing rates in humans have been reported after abrasion combined with meniscal repair, and further confirmed at arthroscopic second looks, although their effectiveness seems to be reduced in the case of tears in poorly vascularized zones [79,80,81].

### 4.3. Microfracture

A lower failure rate of a meniscal repair in case of concomitant ACLr is well documented [43,54,55,56,57,58,82], theoretically due to the release of mesenchymal stromal cells and growth factors from the bone tunnels [72,73], promoting angiogenesis, matrix synthesis, and cell proliferation [83,84,85]. Microfracture of the intercondylar notch aims to simulate this mechanism by creating holes that penetrate the subchondral bone, releasing marrow elements into the joint. Several studies have demonstrated the efficacy and safety of microfractures as an adjunctive technique to meniscal repair [86,87,88], with some authors even speculating on an efficacy superimposed on that obtained by concomitant ACLr [89]. A level I randomized controlled trial by Kaminski et al. demonstrated a 100% healing rate in 23 patients after vertical complete meniscal lesion repair when 6 to 7 microfracture holes into the lateral aspect of the intercondylar notch were performed using a special device [90]. A recent systematic review showed that bone marrow stimulation reduces the likelihood of failure in patients undergoing isolated meniscal repair, although it does not lead to improvements in knee symptom scores [91]. However, clinical results remain contradictory [92], thus questioning its broader applicability [93].

### 4.4. Platelet-Rich Plasma

Different protocols for platelet-rich plasma (PRP) use exist, inevitably affecting results as PRP can be used in single or multiple administrations, in the joint or directly inside the repaired meniscal lesion, as well as on biological supports such as scaffolds or clots. Various preparation techniques lead to multiple types of qualitatively different products. In vitro and in vivo studies support the benefits of PRP augmentation to meniscal repair [94,95,96,97,98]. This is also supported by human clinical studies [99,100,101,102], although results may be conflicting [103,104]. Despite the difficulties in demonstrating the theoretical preclinical benefits of PRP even in humans [95], Kaminski et al. [105] in their level I RCT demonstrated that injecting PRP into the meniscal lesion after repair improves the rate of healing and clinical outcomes. Otherwise, multiple intra-articular injections of PRP do not appear to have any clinical or healing advantage over its non-use [41]. A recent systematic review and meta-analysis of six comparative studies employing PRP augmentation in conjunction with meniscal repair emphasized the biological power of PRP and supported its use as an adjuvant to meniscal healing [106]. This point was also highlighted by the systematic review of Haunschild et al. [107], which argued that PRP augmentation may have the potential to decrease the failure rate after meniscal repair. However, PRP may not improve knee symptom scores [91], although results from a recent systematic review reported mixed results [92,93].

### 4.5. Fibrin Clot

A fibrin clot (FC) is an autologous derivative of a patient’s peripheral blood. It contains growth factors and cytokines which provide both local chemotactic and mitogenic stimuli for cellular repair, triggering the formation of a fibrous connective tissue scar which remodels over time, potentially healing the meniscal tear [108,109,110,111,112,113]. It has been added to meniscal repair with documented good healing rates and clinical results, even in the case of degenerative lesions [114,115,116,117]. Nakanishi et al. [118] showed that during a two-week observation period, there were no significant differences between an FC and platelet-rich fibrin (PRF) clot in terms of the cumulative amount and pattern of growth factor release. Although FC is cheaper and faster to produce than a PRF clot, its application is more technically demanding, invasive, and time-consuming compared to other simpler techniques such as rasping, trephinations, and microfractures. The superiority of FC over these techniques has not been conclusively demonstrated [77], and a recent systematic review considered the current evidence on FC insufficient to draw definitive conclusions on its actual usefulness [93].

### 4.6. Platelet-Rich Fibrin Clot

In 2006, Choukroun et al. [119] developed a platelet derivative called PRF, which was subsequently used by Narayanaswamy et al. [120] as an alternative technique to FC. Compared to its derivatives, this second-generation PRF theoretically offers the advantage of being richer in platelets, which are slowly released over several weeks directly at the site of the repaired meniscal lesion [121,122,123,124,125,126]. Despite numerous well-documented advantages of PRF over PRP and FC [120,124,126,127], its clinical superiority over other techniques is still debated [118]. Although promising, there is insufficient evidence for definitive conclusions [93]. Further in vivo and high-quality research is necessary to clarify the clinical effectiveness of this novel technique.

**Table 4:** Main characteristics of the most common augmentation techniques for meniscal repair.

Technique	Summary	Biology	Clinical Evidence	References
Trephination	A spinal needle is used arthroscopically to create multiple holes in the inner wall of the meniscus to form vascular channels connecting the vascularized area (red-red zone) with the meniscal lesion.	Redirecting the blood supply by allowing migration of cells and factors that stimulate a healing response and remodeling.	Contradictory: effective in promoting meniscal healing only if combined with other techniques.	[70,71,73,74,113]
Microfracture	Once meniscal repair is complete, the arthroscopic fluid flow is interrupted and a blunt tip is used arthroscopically to perform microfractures in the intercondylar notch, at the origin of the PCL, or in the medial aspect of the lateral femoral condyle. These microfractures penetrate the subchondral bone until bone marrow elements are observed entering the joint through the microfracture holes.	Exposes the joint to bone marrow mesenchymal stromal cells and growth factors such as IGF-1, TGF-β, and several BMPs.	Improved meniscal healing at arthroscopic second looks and enhanced clinical results.	[86,87,88,105]
Abrasions	A rasp or shaver is used arthroscopically on the edges of the meniscal lesion and parameniscal synovium to revitalize the meniscal tissue and stimulate the release of growth factors from the synovial surface.	Promotes the release of IL-1α, TGF-β1, PDGF, and PCNA.	Good results shown by arthroscopic second looks. Less effective with lesions in poorly vascularized zones.	[75,77,78,79,80,81,108]
Fibrin clot	Peripheral blood is taken from the patient. The blood is placed in a sterile specimen cup and then stirred using a frosted glass syringe plunger until the clot is completely formed. Afterwards, it is washed with saline and introduced into the joint.	Composed of blood factors including 5% of platelets that are progressively released in situ.	Satisfactory outcomes with high healing rates despite failures are still present in a small variable percentage. Greater efficacy compared to other techniques is uncertain.	[114,115,116,118]
PRF clot	The patient’s peripheral blood is drawn and placed in a sterile glass tube and centrifuged. After centrifugation, the clot is left to consolidate for 5–10 min. Then, it is removed from the centrifuge and the sterile glass tube and separated between the red clot and white clot junction. Finally, it is introduced into the joint.	Composed by 85–95% of platelets as well as a fibrin matrix polymer, leucocytes, cytokines, and stem cells. Progressive and localized release of cytokines, antimicrobial peptides, cells, and growth factors.	The theoretical advantages are numerous, although adequate clinical studies demonstrating this are lacking. Theoretical superiority over fibrin clot is debated.	[119,120,124,126,127]
PRP	Single or multiple intra-articular or intra-lesion injections of PRP.	Introduces growth factors (PDGF, VEGF, TGF-β1) to promote chemotaxis, angiogenesis, collagen matrix synthesis, and cell proliferation, eventually enhancing meniscal healing.	It is contradictory as it depends on the mode of use and the characteristics of the PRP. Intra-lesion injection appears more effective than intra-articular administration.	[94,95,96,97,98,99,100,101,102,105]

Abbreviations: BMP = bone morphogenetic proteins; IGF-1 = insulin-like growth factor-1; IL = interleukin; PCL = posterior cruciate ligament, PCNA = proliferating cell nuclear antigen; PDGF = platelet-derived growth factor; PRF = platelet-rich fibrin; PRP = platelet-rich plasma; TGF-β = transforming growth factor β, VEGF = vascular endothelial growth factor.

### 4.7. Future Perspectives

Although several biological augmentation techniques have been introduced to improve meniscal healing rates, the overall quality of the evidence remains low. The strategies employed are significantly heterogeneous, and the underlying mechanisms are not yet fully understood [69,80,83]. Nonetheless, novel approaches, including both intraarticular and extraarticular systemic strategies, are under investigation [91,92,93]. Among these, stimulating endogenous irisin release could potentially enhance meniscal repair. Irisin, a myokine physiologically released by skeletal muscle during physical exercise, has been shown in previous studies to exert both paracrine and endocrine effects on various musculoskeletal tissues, including cartilage [128], tendon [129], and intervertebral disc [130]. By potentially stimulating meniscal healing, dedicated physical therapy protocols designed to increase endogenous irisin production could be implemented following postoperative repair. However, these theories remain speculative and require preclinical validation.

## 5. Conclusions

The current trend favors meniscal injury repair due to its numerous advantages over meniscectomy, especially in the medium and long term. However, the relatively high failure rate and the challenge of repairing biologically disadvantaged meniscal injuries remain significant issues. The biological augmentation of meniscal repair through various techniques represents a safe and effective option with proven clinical benefits. This approach could reduce the failure rate and expand the indications for meniscal repair. However, conclusive evidence from standardized, high-quality research is needed to establish the definitive efficacy of these biological augmentation techniques.

## Figures and Tables

**Table 1:** Studies comparing outcomes of meniscectomy versus meniscal repair.

Study (Year)	Study Design (LOE)	Mean Age or Range (Years)	Mean Follow-Up (Months)	Tear Location	PROMs				Radiological Outcomes	Failure Rate	Reoperation Rate
					IKDC	Lysholm Score	TAS	KOOS			
Xu et al. [47] (2015)	Meta-analysis (III)	22–33	84	NS	MR: ↑	MR: ↑	MR: ↑	NS	NS	MR: ↓	NS
Bottomley et al. [48] (2023)	Retrospective study (III)	MR: 47 ME: 61	MR: 57	Mixed	MR: ↑	MR: ↑	MR: ↑	MR: ↑	NS	NS	NS
			ME: 47								
Ro et al. [49] (2020)	Meta-analysis (IV)	MR: 54 ME: 56	MR: 33.5	MMPRT	NS	MR: ↑	MR = ME	NS	MR showed lower rates of OA progression	NS	MR 4.2%
			ME: 47.2								ME: 32%
Duethman et al. [15] (2021)	Retrospective study (III)	17.4	100	LM	MR: ↑	NS	NS	NS	ME showed higher rates of symptomatic OA	MR: 27% ME: 27%	MR: 23% ME: 18%
Paxton et al. [43] (2011)	Systematic review (IV)	MR: 26.0–46.8 ME: 15.3–44.2	>120	Mixed	NS	MR = ME	NS	NS	MR showed lower rates of OA progression	NS	MR: 16.5% (0–48 months), 20.7% (>120 months)
											ME: 1.4% (0–48 months), 3.9% (>120 months)
D’Ambrosi et al. [50] (2022)	Systematic review (IV)	MR: 22.1 ME: 23.3	NS	NS	NS	NS	NS	NS	NS	NS	MR: 17% (LM 25%, MM 25%, 50% NS)
											ME: 3.7% (LM 83%, MM 17%)
Migliorini et al. [51] (2023)	Meta-analysis (III)	37.6	63	NS	MR = ME	ME: ↑	NS	NS	MR showed lower rates of OA progression	No differences	MR: 14%
Stein et al. [52] (2010)	Cohort study (III)	MR: 31.3 ME: 32.5	40 (mid-term	MM	NS	NS	NS	NS	ME showed higher OA progression in the long-term	NS	ME: 10%
			104 (long-term)		NS	MR = ME	NS	NS			
Shieh et al. [42] (2016)	Case–control study (III)	15.3	40	NS	NS	NS	NS	NS	NS	NS	MR: 18%
											ME: 7%

Upward arrows indicate the superiority of MR with regards to the investigated outcomes, whereas the downward arrow indicates a lower failure rate in the MR group. Abbreviations: IKDC = International Knee Documentation Committee; KOOS = Knee injury and Osteoarthritis Outcome Score; LM = lateral meniscus, LOE = level of evidence; ME = meniscectomy; MM = medial meniscus; MMPRT = medial meniscus posterior root tears; MR = meniscal repair; NS = not specified, TAS = Tegner activity score.

**Table 2:** Systematic reviews and meta-analyses reporting reoperation or overall failure rates of meniscal repair.

Study (Year)	Study Design (LOE)	Mean Follow-Up (Months)	Tear Localization	Overall Failure Rate	Reoperation Rate
Schweizer et al. [65] (2021)	Meta-analysis (III)	86	MM and LM	19.1%	MM 24.4%, LM 19.5%
Schweizer et al. [59] (2023)	Meta-analysis (IV)	24–60	MM and LM	14.8% (MM 10.8%, LM 6.1%)	NS
Ro et al. [49] (2020)	Meta-analysis (IV)	33.5	MMPRT	NS	4.2%
Migliorini et al. [51] (2023)	Meta-analysis (IV)	63	NS	NS	14%
Migliorini et al. [51] (2023)	Systematic review (III)	67	MM and LM	5.9%	1.1%
Blanchard et al. [66] (2020)	Systematic review (IV)	40.5	MM and LM		12.4%
Dai et al. [67] (2021)	Systematic review (IV)	19–58 (range)	MM and LM	MM: 15%; LM 14%	NS
D’Ambrosi et al. [50] (2022)	Systematic review (IV)	NS	NS	NS	17% (LM 25%, MM 25%, 50% NS)
Paxton et al. [43] (2011)	Systematic review (IV)	>120	MM and LM	NS	16.5% (0–48 months), 20.7% (>120 months)

Abbreviations: LM = lateral meniscus, LOE = level of evidence; MM = medial meniscus; MMPRT = medial meniscus posterior root tears, NS = not specified.

**Table 3:** Level III and IV clinical studies reporting reoperation or overall failure rates of meniscal repair.

Study (Year)	Study Design (LOE)	Mean Follow-Up (Months)	Tear Location	Overall Failure Rate	Reoperation Rate
Hagmeijer et al. [53] (2019)	Case series (IV)	210	MM and LM	42% (complex tears 80%; bucket-handle tears 47%; simple tears 18.2%)	36%
Ronnblad et al. [54] (2020)	Case–control study (III)	96	MM and LM	22.5%	MM 28.3%, LM 11.7%
Abdelkafy et al. [68] (2005)	Retrospective study (III)	139		12%	
Duethman et al. [15] (2021)	Retrospective study (III)	100	LM	27%	23%
Shieh et al. [42] (2016)	Case–control study (III)	40			18%

Abbreviations: LM = lateral meniscus, LOE = level of evidence; MM = medial meniscus.

## Data Availability

Not applicable.

## References

[1] Kim S., Bosque J., Meehan J.P., Jamali A., Marder R. (2011). Increase in Outpatient Knee Arthroscopy in the United States: A Comparison of National Surveys of Ambulatory Surgery, 1996 and 2006. J. Bone Jt. Surg..

[2] Herrlin S., Hållander M., Wange P., Weidenhielm L., Werner S. (2007). Arthroscopic or Conservative Treatment of Degenerative Medial Meniscal Tears: A Prospective Randomised Trial. Knee Surg. Sports Traumatol. Arthrosc..

[3] Thorlund J.B., Hare K.B., Lohmander L.S. (2014). Large Increase in Arthroscopic Meniscus Surgery in the Middle-Aged and Older Population in Denmark from 2000 to 2011. Acta Orthop..

[4] Fabricant P.D., Jokl P. (2007). Surgical Outcomes After Arthroscopic Partial Meniscectomy. J. Am. Acad. Orthop. Surg..

[5] Burks R.T., Metcalf M.H., Metcalf R.W. (1997). Fifteen-Year Follow-up of Arthroscopic Partial Meniscectomy. Arthrosc. J. Arthrosc. Relat. Surg..

[6] Katz J.N., Brophy R.H., Chaisson C.E., De Chaves L., Cole B.J., Dahm D.L., Donnell-Fink L.A., Guermazi A., Haas A.K., Jones M.H. (2013). Surgery versus Physical Therapy for Meniscal Tear and Osteoarthritis. N. Engl. J. Med..

[7] Herrlin S.V., Wange P.O., Lapidus G., Hållander M., Werner S., Weidenhielm L. (2013). Is Arthroscopic Surgery Beneficial in Treating Non-Traumatic, Degenerative Medial Meniscal Tears? A Five Year Follow-Up. Knee Surg. Sports Traumatol. Arthrosc..

[8] Kirkley A., Birmingham T.B., Litchfield R.B., Giffin J.R., Willits K.R., Wong C.J., Feagan B.G., Donner A., Griffin S.H., D’Ascanio L.M. (2008). A Randomized Trial of Arthroscopic Surgery for Osteoarthritis of the Knee. N. Engl. J. Med..

[9] Sihvonen R., Paavola M., Malmivaara A., Itälä A., Joukainen A., Nurmi H., Kalske J., Järvinen T.L.N. (2013). Arthroscopic Partial Meniscectomy versus Sham Surgery for a Degenerative Meniscal Tear. N. Engl. J. Med..

[10] Beaufils P., Hulet C., Dhénain M., Nizard R., Nourissat G., Pujol N. (2009). Clinical Practice Guidelines for the Management of Meniscal Lesions and Isolated Lesions of the Anterior Cruciate Ligament of the Knee in Adults. Orthop. Traumatol. Surg. Res..

[11] Sihvonen R., Englund M., Turkiewicz A., Järvinen T.L.N., Finnish Degenerative Meniscal Lesion Study Group (2016). Mechanical Symptoms and Arthroscopic Partial Meniscectomy in Patients with Degenerative Meniscus Tear: A Secondary Analysis of a Randomized Trial. Ann. Intern. Med..

[12] Kim J.-Y., Bin S.-I., Kim J.-M., Lee B.-S., Oh S.-M., Cho W.-J., Lee J.-H. (2020). Partial Meniscectomy Provides the Favorable Outcomes for Symptomatic Medial Meniscus Tear with an Intact Posterior Root. Knee Surg. Sports Traumatol. Arthrosc..

[13] Mariani P.P., Garofalo R., Margheritini F. (2008). Chondrolysis after Partial Lateral Meniscectomy in Athletes. Knee Surg. Sports Traumatol. Arthr..

[14] Pioger C., Saithna A., Kandhari V., Thaunat M., Vieira T.D., Freychet B., Franck F., Sonnery-Cottet B. (2021). Risk Factors for Rapid Chondrolysis After Partial Lateral Meniscectomy: A Scoping Review of the Literature. Orthop. J. Sports Med..

[15] Duethman N.C., Wilbur R.R., Song B.M., Stuart M.J., Levy B.A., Camp C.L., Krych A.J. (2021). Lateral Meniscal Tears in Young Patients: A Comparison of Meniscectomy and Surgical Repair. Orthop. J. Sports Med..

[16] Lee B.-S., Bin S.-I., Kim J.-M., Park M.-H., Lee S.-M., Bae K.-H. (2019). Partial Meniscectomy for Degenerative Medial Meniscal Root Tears Shows Favorable Outcomes in Well-Aligned, Nonarthritic Knees. Am. J. Sports Med..

[17] Kim C., Bin S.-I., Kim J.-M., Lee B.-S., Kim T.-H. (2020). Progression of Radiographic Osteoarthritis after Partial Meniscectomy in Degenerative Medial Meniscal Posterior Root Tears Was Greater in Varus- than in Neutral-Aligned Knees: A Minimum 5-Year Follow-Up. Knee Surg. Sports Traumatol. Arthrosc..

[18] Kluczynski M.A., Marzo J.M., Wind W.M., Fineberg M.S., Bernas G.A., Rauh M.A., Zhou Z., Zhao J., Bisson L.J. (2017). The Effect of Body Mass Index on Clinical Outcomes in Patients Without Radiographic Evidence of Degenerative Joint Disease After Arthroscopic Partial Meniscectomy. Arthrosc. J. Arthrosc. Relat. Surg..

[19] Feeley B.T., Lau B.C. (2018). Biomechanics and Clinical Outcomes of Partial Meniscectomy. J. Am. Acad. Orthop. Surg..

[20] Paradowski P.T., Lohmander L.S., Englund M. (2016). Osteoarthritis of the Knee after Meniscal Resection: Long Term Radiographic Evaluation of Disease Progression. Osteoarthr. Cartil..

[21] Thorlund J.B., Holsgaard-Larsen A., Creaby M.W., Jørgensen G.M., Nissen N., Englund M., Lohmander L.S. (2016). Changes in Knee Joint Load Indices from before to 12 Months after Arthroscopic Partial Meniscectomy: A Prospective Cohort Study. Osteoarthr. Cartil..

[22] Brophy R.H., Gray B.L., Nunley R.M., Barrack R.L., Clohisy J.C. (2014). Total Knee Arthroplasty After Previous Knee Surgery: Expected Interval and the Effect on Patient Age. J. Bone Jt. Surg..

[23] Aprato A., Sordo L., Costantino A., Sabatini L., Barberis L., Testa D., Massè A. (2021). Outcomes at 20 Years after Meniscectomy in Young Patients. Knee.

[24] Aprato A., Sordo L., Costantino A., Sabatini L., Barberis L., Testa D., Massè A. (2021). Outcomes at 20 Years After Meniscectomy in Patients Aged 50 to 70 Years. Arthrosc. J. Arthrosc. Relat. Surg..

[25] https://www.canada.ca/en/public-health/services/reports-publications/health-promotion-chronic-disease-prevention-canada-research-policy-practice/vol-31-no-3-2011/report-summary-life-with-arthritis-canada-personal-public-health-challenge.html.

[26] Musahl V., Citak M., O’Loughlin P.F., Choi D., Bedi A., Pearle A.D. (2010). The Effect of Medial Versus Lateral Meniscectomy on the Stability of the Anterior Cruciate Ligament-Deficient Knee. Am. J. Sports Med..

[27] Hasan J., Fisher J., Ingham E. (2014). Current Strategies in Meniscal Regeneration. J. Biomed. Mater. Res..

[28] Brown M.J., Farrell J.P., Kluczynski M.A., Marzo J.M. (2016). Biomechanical Effects of a Horizontal Medial Meniscal Tear and Subsequent Leaflet Resection. Am. J. Sports Med..

[29] Koh J.L., Yi S.J., Ren Y., Zimmerman T.A., Zhang L.-Q. (2016). Tibiofemoral Contact Mechanics with Horizontal Cleavage Tear and Resection of the Medial Meniscus in the Human Knee. J. Bone Jt. Surg..

[30] Goyal K.S., Pan T.J., Tran D., Dumpe S.C., Zhang X., Harner C.D. (2014). Vertical Tears of the Lateral Meniscus: Effects on In Vitro Tibiofemoral Joint Mechanics. Orthop. J. Sports Med..

[31] Ode G.E., Van Thiel G.S., McArthur S.A., Dishkin-Paset J., Leurgans S.E., Shewman E.F., Wang V.M., Cole B.J. (2012). Effects of Serial Sectioning and Repair of Radial Tears in the Lateral Meniscus. Am. J. Sports Med..

[32] Bedi A., Kelly N., Baad M., Fox A.J.S., Ma Y., Warren R.F., Maher S.A. (2012). Dynamic Contact Mechanics of Radial Tears of the Lateral Meniscus: Implications for Treatment. Arthrosc. J. Arthrosc. Relat. Surg..

[33] Sturnieks D.L., Besier T.F., Mills P.M., Ackland T.R., Maguire K.F., Stachowiak G.W., Podsiadlo P., Lloyd D.G. (2008). Knee Joint Biomechanics Following Arthroscopic Partial Meniscectomy. J. Orthop. Res..

[34] Hall M., Wrigley T.V., Metcalf B.R., Hinman R.S., Cicuttini F.M., Dempsey A.R., Mills P.M., Lloyd D.G., Bennell K.L. (2015). Mechanisms Underpinning the Peak Knee Flexion Moment Increase over 2-Years Following Arthroscopic Partial Meniscectomy. Clin. Biomech..

[35] Beamer B.S., Walley K.C., Okajima S., Manoukian O.S., Perez-Viloria M., DeAngelis J.P., Ramappa A.J., Nazarian A. (2017). Changes in Contact Area in Meniscus Horizontal Cleavage Tears Subjected to Repair and Resection. Arthrosc. J. Arthrosc. Relat. Surg..

[36] Krause W.R., Pope M.H., Johnson R.J., Wilder D.G. (1976). Mechanical Changes in the Knee after Meniscectomy. J Bone Jt. Surg Am.

[37] Barber F.A., McGarry J.E. (2007). Meniscal Repair Techniques. Sports Med. Arthrosc. Rev..

[38] Beaufils P., Pujol N. (2018). Meniscal Repair: Technique. Orthop. Traumatol. Surg. Res..

[39] Rothermel S.D., Smuin D., Dhawan A. (2018). Are Outcomes After Meniscal Repair Age Dependent? A Systematic Review. Arthrosc. J. Arthrosc. Relat. Surg..

[40] Krych A.J., McIntosh A.L., Voll A.E., Stuart M.J., Dahm D.L. (2008). Arthroscopic Repair of Isolated Meniscal Tears in Patients 18 Years and Younger. Am. J. Sports Med..

[41] Yang C.-P., Hung K.-T., Weng C.-J., Chen A.C.-Y., Hsu K.-Y., Chan Y.-S. (2021). Clinical Outcomes of Meniscus Repair with or without Multiple Intra-Articular Injections of Platelet Rich Plasma after Surgery. J. Clin. Med..

[42] Shieh A.K., Edmonds E.W., Pennock A.T. (2016). Revision Meniscal Surgery in Children and Adolescents: Risk Factors and Mechanisms for Failure and Subsequent Management. Am. J. Sports Med..

[43] Paxton E.S., Stock M.V., Brophy R.H. (2011). Meniscal Repair Versus Partial Meniscectomy: A Systematic Review Comparing Reoperation Rates and Clinical Outcomes. Arthrosc. J. Arthrosc. Relat. Surg..

[44] Chahla J., Kennedy N.I., Geeslin A.G., Moatshe G., Cinque M.E., DePhillipo N.N., LaPrade R.F. (2017). Meniscal Repair with Fibrin Clot Augmentation. Arthrosc. Tech..

[45] Krych A.J., Reardon P., Sousa P., Levy B.A., Dahm D.L., Stuart M.J. (2016). Clinical Outcomes After Revision Meniscus Repair. Arthrosc. J. Arthrosc. Relat. Surg..

[46] Ozeki N., Seil R., Krych A.J., Koga H. (2021). Surgical Treatment of Complex Meniscus Tear and Disease: State of the Art. J. ISAKOS.

[47] Xu C., Zhao J. (2015). A Meta-Analysis Comparing Meniscal Repair with Meniscectomy in the Treatment of Meniscal Tears: The More Meniscus, the Better Outcome?. Knee Surg. Sports Traumatol. Arthrosc..

[48] Bottomley J., Al-Dadah O. (2023). Arthroscopic Meniscectomy vs Meniscal Repair: Comparison of Clinical Outcomes. Cureus.

[49] Ro K.-H., Kim J.-H., Heo J.-W., Lee D.-H. (2020). Clinical and Radiological Outcomes of Meniscal Repair Versus Partial Meniscectomy for Medial Meniscus Root Tears: A Systematic Review and Meta-Analysis. Orthop. J. Sports Med..

[50] D’Ambrosi R., Meena A., Raj A., Ursino N., Mangiavini L., Herbort M., Fink C. (2023). In Elite Athletes with Meniscal Injuries, Always Repair the Lateral, Think about the Medial! A Systematic Review. Knee Surg Sports Traumatol. Arthrosc..

[51] Migliorini F., Schäfer L., Bell A., Weber C.D., Vecchio G., Maffulli N. (2023). Meniscectomy Is Associated with a Higher Rate of Osteoarthritis Compared to Meniscal Repair Following Acute Tears: A Meta-analysis. Knee Surg. Sports Traumatol. Arthrosc..

[52] Stein T., Mehling A.P., Welsch F., Von Eisenhart-Rothe R., Jäger A. (2010). Long-Term Outcome After Arthroscopic Meniscal Repair Versus Arthroscopic Partial Meniscectomy for Traumatic Meniscal Tears. Am. J. Sports Med..

[53] Hagmeijer M.H., Kennedy N.I., Tagliero A.J., Levy B.A., Stuart M.J., Saris D.B.F., Dahm D.L., Krych A.J. (2019). Long-Term Results After Repair of Isolated Meniscal Tears Among Patients Aged 18 Years and Younger: An 18-Year Follow-up Study. Am. J. Sports Med..

[54] Ronnblad E., Barenius B., Engstrom B., Eriksson K. (2020). Predictive Factors for Failure of Meniscal Repair: A Retrospective Dual-Center Analysis of 918 Consecutive Cases. Orthop. J. Sports Med..

[55] Tenuta J.J., Arciero R.A. (1994). Arthroscopic Evaluation of Meniscal Repairs: Factors That Effect Healing. Am. J. Sports Med..

[56] Cannon W.D., Vittori J.M. (1992). The Incidence of Healing in Arthroscopic Meniscal Repairs in Anterior Cruciate Ligament-Reconstructed Knees versus Stable Knees. Am. J. Sports Med..

[57] Abrams G.D., Frank R.M., Gupta A.K., Harris J.D., McCormick F.M., Cole B.J. (2013). Trends in Meniscus Repair and Meniscectomy in the United States, 2005–2011. Am. J. Sports Med..

[58] Jin Hwan A., Lee Y.S., Yoo J.C., Chang M.J., Koh K.H., Kim M.H. (2010). Clinical and Second-Look Arthroscopic Evaluation of Repaired Medial Meniscus in Anterior Cruciate Ligament—Reconstructed Knees. Am. J. Sports Med..

[59] Schweizer C., Hanreich C., Tscholl P.M., Blatter S., Windhager R., Waldstein W. (2024). Meniscal Repair Outcome in 3829 Patients with a Minimum Follow-up from 2 Years up to 5 Years: A Meta-Analysis on the Overall Failure Rate and Factors Influencing Failure. Am. J. Sports Med..

[60] Barrett G. (1998). Clinical Results of Meniscus Repair in Patients 40 Years and Older. Arthrosc. J. Arthrosc. Relat. Surg..

[61] Kise N.J., Drogset J.O., Ekeland A., Sivertsen E.A., Heir S. (2015). All-inside Suture Device Is Superior to Meniscal Arrows in Meniscal Repair: A Prospective Randomized Multicenter Clinical Trial with 2-Year Follow-Up. Knee Surg. Sports Traumatol. Arthrosc..

[62] Steadman J.R., Matheny L.M., Singleton S.B., Johnson N.S., Rodkey W.G., Crespo B., Briggs K.K. (2015). Meniscus Suture Repair: Minimum 10-Year Outcomes in Patients Younger Than 40 Years Compared with Patients 40 and Older. Am. J. Sports Med..

[63] Wouters D.B. (2023). Repair of a Meniscus Tear within 3 Weeks after Trauma Significantly Reduces the Likelihood of a Recurrent Tear Compared with Later Repairs. Knee Surg. Sports Traumatol. Arthrosc..

[64] O’Donnell K., Freedman K.B., Tjoumakaris F.P. (2017). Rehabilitation Protocols After Isolated Meniscal Repair: A Systematic Review. Am. J. Sports Med..

[65] Schweizer C., Hanreich C., Tscholl P.M., Ristl R., Apprich S., Windhager R., Waldstein W. (2022). Nineteen Percent of Meniscus Repairs Are Being Revised and Failures Frequently Occur after the Second Postoperative Year: A Systematic Review and Meta-Analysis with a Minimum Follow-up of 5 Years. Knee Surg. Sports Traumatol. Arthrosc..

[66] Blanchard E.R., Hadley C.J., Wicks E.D., Emper W., Cohen S.B. (2020). Return to Play After Isolated Meniscal Repairs in Athletes: A Systematic Review. Orthop. J. Sports Med..

[67] Dai W., Leng X., Wang J., Hu X., Ao Y. (2021). Second-Look Arthroscopic Evaluation of Healing Rates After Arthroscopic Repair of Meniscal Tears: A Systematic Review and Meta-Analysis. Orthop. J. Sports Med..

[68] Abdelkafy A., Aigner N., Zada M., Elghoul Y., Abdelsadek H., Landsiedl F. (2007). Two to Nineteen Years Follow-up of Arthroscopic Meniscal Repair Using the Outside-in Technique: A Retrospective Study. Arch. Orthop. Trauma Surg..

[69] Longo U.G., Campi S., Romeo G., Spiezia F., Maffulli N., Denaro V. (2012). Biological Strategies to Enhance Healing of the Avascular Area of the Meniscus. Stem Cells Int..

[70] Cook J.L., Fox D.B. (2007). A Novel Bioabsorbable Conduit Augments Healing of Avascular Meniscal Tears in a Dog Model. Am. J. Sports Med..

[71] Zhang Z., Tu K., Xu Y., Zhang W., Liu Z., Ou S. (1988). Treatment of Longitudinal Injuries in Avascular Area of Meniscus in Dogs by Trephination. Arthrosc. J. Arthrosc. Relat. Surg..

[72] Forriol F., Longo U., Duart J., Ripalda P., Vaquero J., Loppini M., Romeo G., Campi S., Khan W., Muda A. (2014). VEGF, BMP-7, Matrigel^TM^, Hyaluronic Acid, In Vitro Cultured Chondrocytes and Trephination for Healing of the Avascular Portion of the Meniscus. An Experimental Study in Sheep. Curr. Stem Cell Res. Ther..

[73] Zhang Z., Arnold J.A. (1996). Trephination and Suturing of Avascular Meniscal Tears: A Clinical Study of the Trephination Procedure. Arthrosc. J. Arthrosc. Relat. Surg..

[74] Fox J.M., Rintz K.G., Ferkel R.D. (1993). Trephination of Incomplete Meniscal Tears. Arthrosc. J. Arthrosc. Relat. Surg..

[75] Ochi M., Uchio Y., Okuda K., Shu N., Yamaguchi H., Sakai Y. (2001). Expression of Cytokines after Meniscal Rasping to Promote Meniscal Healing. Arthrosc. J. Arthrosc. Relat. Surg..

[76] Arnoczky S.P., Warren R.F. (1983). The Microvasculature of the Meniscus and Its Response to Injury: An Experimental Study in the Dog. Am. J. Sports Med..

[77] Ritchie J.R., Miller M.D., Bents R.T., Smith D.K. (1998). Meniscal Repair in the Goat Model. Am. J. Sports Med..

[78] Okuda K., Ochi M., Shu N., Uchio Y. (1999). Meniscal Rasping for Repair of Meniscal Tear in the Avascular Zone. Arthrosc. J. Arthrosc. Relat. Surg..

[79] Uchio Y., Ochi M., Adachi N., Kawasaki K., Iwasa J. (2003). Results of Rasping of Meniscal Tears with and without Anterior Cruciate Ligament Injury as Evaluated by Second-Look Arthroscopy. Arthrosc. J. Arthrosc. Relat. Surg..

[80] Ghazi Zadeh L., Chevrier A., Farr J., Rodeo S., Buschmann M. (2018). Augmentation Techniques for Meniscus Repair. J. Knee Surg..

[81] Tetik O., Kocabey Y., Johnson D.L. (2002). Synovial Abrasion for Isolated, Partial Thickness, Undersurface, Medial Meniscus Tears. Orthopedics.

[82] Krych A.J., Pitts R.T., Dajani K.A., Stuart M.J., Levy B.A., Dahm D.L. (2010). Surgical Repair of Meniscal Tears with Concomitant Anterior Cruciate Ligament Reconstruction in Patients 18 Years and Younger. Am. J. Sports Med..

[83] LaPrade C., James E., LaPrade R., Engebretsen L. (2014). How Should We Evaluate Outcomes for Use of Biologics in the Knee?. J. Knee Surg..

[84] Hutchinson I.D., Moran C.J., Potter H.G., Warren R.F., Rodeo S.A. (2014). Restoration of the Meniscus: Form and Function. Am. J. Sports Med..

[85] Martínez C.E., Smith P.C., Palma Alvarado V.A. (2015). The Influence of Platelet-Derived Products on Angiogenesis and Tissue Repair: A Concise Update. Front. Physiol..

[86] Howarth W.R., Brochard K., Campbell S.E., Grogan B.F. (2016). Effect of Microfracture on Meniscal Tear Healing in a Goat (*Capra Hircus*) Model. Orthopedics.

[87] Ahn J.-H., Kwon O.-J., Nam T.-S. (2015). Arthroscopic Repair of Horizontal Meniscal Cleavage Tears with Marrow-Stimulating Technique. Arthrosc. J. Arthrosc. Relat. Surg..

[88] Freedman K.B., Nho S.J., Cole B.J. (2003). Marrow Stimulating Technique to Augment Meniscus Repair. Arthrosc. J. Arthrosc. Relat. Surg..

[89] Dean C.S., Chahla J., Matheny L.M., Mitchell J.J., LaPrade R.F. (2017). Outcomes After Biologically Augmented Isolated Meniscal Repair with Marrow Venting Are Comparable with Those After Meniscal Repair With Concomitant Anterior Cruciate Ligament Reconstruction. Am. J. Sports Med..

[90] Kaminski R., Kulinski K., Kozar-Kaminska K., Wasko M.K., Langner M., Pomianowski S. (2019). Repair Augmentation of Unstable, Complete Vertical Meniscal Tears with Bone Marrow Venting Procedure: A Prospective, Randomized, Double-Blind, Parallel-Group, Placebo-Controlled Study. Arthrosc. J. Arthrosc. Relat. Surg..

[91] Blough C.L., Bobba C.M., DiBartola A.C., Everhart J.S., Magnussen R.A., Kaeding C., Flanigan D.C. (2023). Biologic Augmentation during Meniscal Repair. J. Knee Surg..

[92] Keller R.E., O’Donnell E.A., Medina G.I.S., Linderman S.E., Cheng T.T.W., Sabbag O.D., Oh L.S. (2022). Biological Augmentation of Meniscal Repair: A Systematic Review. Knee Surg. Sports Traumatol. Arthrosc..

[93] Chen K., Aggarwal S., Baker H., Athiviraham A. (2024). Biologic Augmentation of Isolated Meniscal Repair. Curr. Rev. Musculoskelet. Med..

[94] Ishida K., Kuroda R., Miwa M., Tabata Y., Hokugo A., Kawamoto T., Sasaki K., Doita M., Kurosaka M. (2007). The Regenerative Effects of Platelet-Rich Plasma on Meniscal Cells In Vitro and Its In Vivo Application with Biodegradable Gelatin Hydrogel. Tissue Eng..

[95] Zellner J., Mueller M., Berner A., Dienstknecht T., Kujat R., Nerlich M., Hennemann B., Koller M., Prantl L., Angele M. (2010). Role of Mesenchymal Stem Cells in Tissue Engineering of Meniscus. J. Biomed. Mater. Res..

[96] Spindler K.P., Mayes C.E., Miller R.R., Imro A.K., Davidson J.M. (1995). Regional Mitogenic Response of the Meniscus to Platelet-derived Growth Factor (PDGF-AB). J. Orthop. Res..

[97] Xu H., Zou X., Xia P., Aboudi M.A.K., Chen R., Huang H. (2020). Differential Effects of Platelets Selectively Activated by Protease-Activated Receptors on Meniscal Cells. Am. J. Sports Med..

[98] Wei L.-C., Gao S.-G., Xu M., Jiang W., Tian J., Lei G.-H. (2012). A Novel Hypothesis: The Application of Platelet-Rich Plasma Can Promote the Clinical Healing of White-White Meniscal Tears. Med. Sci. Monit..

[99] Pujol N., Salle De Chou E., Boisrenoult P., Beaufils P. (2015). Platelet-Rich Plasma for Open Meniscal Repair in Young Patients: Any Benefit?. Knee Surg. Sports Traumatol. Arthrosc..

[100] Sochacki K.R., Safran M.R., Abrams G.D., Donahue J., Chu C., Sherman S.L. (2020). Platelet-Rich Plasma Augmentation for Isolated Arthroscopic Meniscal Repairs Leads to Significantly Lower Failure Rates: A Systematic Review of Comparative Studies. Orthop. J. Sports Med..

[101] Kemmochi M., Sasaki S., Takahashi M., Nishimura T., Aizawa C., Kikuchi J. (2018). The Use of Platelet-Rich Fibrin with Platelet-Rich Plasma Support Meniscal Repair Surgery. J. Orthop..

[102] Dai W.-L., Zhang H., Lin Z.-M., Shi Z.-J., Wang J. (2019). Efficacy of Platelet-Rich Plasma in Arthroscopic Repair for Discoid Lateral Meniscus Tears. BMC Musculoskelet. Disord..

[103] Griffin J.W., Hadeed M.M., Werner B.C., Diduch D.R., Carson E.W., Miller M.D. (2015). Platelet-Rich Plasma in Meniscal Repair: Does Augmentation Improve Surgical Outcomes?. Clin. Orthop. Relat. Res..

[104] Everhart J.S., Cavendish P.A., Eikenberry A., Magnussen R.A., Kaeding C.C., Flanigan D.C. (2019). Platelet-Rich Plasma Reduces Failure Risk for Isolated Meniscal Repairs but Provides No Benefit for Meniscal Repairs with Anterior Cruciate Ligament Reconstruction. Am. J. Sports Med..

[105] Kaminski R., Kulinski K., Kozar-Kaminska K., Wielgus M., Langner M., Wasko M.K., Kowalczewski J., Pomianowski S. (2018). A Prospective, Randomized, Double-Blind, Parallel-Group, Placebo-Controlled Study Evaluating Meniscal Healing, Clinical Outcomes, and Safety in Patients Undergoing Meniscal Repair of Unstable, Complete Vertical Meniscal Tears (Bucket Handle) Augmented with Platelet-Rich Plasma. BioMed Res. Int..

[106] Zaffagnini S., Poggi A., Reale D., Andriolo L., Flanigan D.C., Filardo G. (2021). Biologic Augmentation Reduces the Failure Rate of Meniscal Repair: A Systematic Review and Meta-Analysis. Orthop. J. Sports Med..

[107] Haunschild E.D., Huddleston H.P., Chahla J., Gilat R., Cole B.J., Yanke A.B. (2020). Platelet-Rich Plasma Augmentation in Meniscal Repair Surgery: A Systematic Review of Comparative Studies. Arthrosc. J. Arthrosc. Relat. Surg..

[108] Arnoczky S.P., Warren R.F., Spivak J.M. (1988). Meniscal Repair Using an Exogenous Fibrin Clot. An Experimental Study in Dogs. J. Bone Jt. Surg. Am..

[109] Henning C.E., Lynch M.A., Yearout K.M., Vequist S.W., Stallbaumer R.J., Decker K.A. (1990). Arthroscopic Meniscal Repair Using an Exogenous Fibrin Clot. Clin. Orthop. Relat. Res..

[110] Ishimura M., Tamai S., Fujisawa Y. (1991). Arthroscopic Meniscal Repair with Fibrin Glue. Arthrosc. J. Arthrosc. Relat. Surg..

[111] Van Trommel M., Simonian P., Potter H., Wickiewicz T. (1998). Arthroscopic Meniscal Repair with Fibrin Clot of Complete Radial Tears of the Lateral Meniscus in the Avascular Zone. Arthrosc. J. Arthrosc. Relat. Surg..

[112] Forriol F., Denaro V., Denaro L., Longo U.G., Taira H. (2010). Bone Lengthening Osteogenesis, a Combination of Intramembranous and Endochondral Ossification: An Experimental Study in Sheep. Strateg. Trauma Limb Reconstr..

[113] Forriol F., Longo U.G., Concejo C., Ripalda P., Maffulli N., Denaro V. (2009). Platelet-Rich Plasma, rhOP-1® (rhBMP-7) and Frozen Rib Allograft for the Reconstruction of Bony Mandibular Defects in Sheep. A Pilot Experimental Study. Injury.

[114] Kamimura T., Kimura M. (2014). Meniscal Repair of Degenerative Horizontal Cleavage Tears Using Fibrin Clots: Clinical and Arthroscopic Outcomes in 10 Cases. Orthop. J. Sports Med..

[115] Nakayama H., Kanto R., Kambara S., Iseki T., Onishi S., Yoshiya S. (2020). Successful Treatment of Degenerative Medial Meniscal Tears in Well-Aligned Knees with Fibrin Clot Implantation. Knee Surg. Sports Traumatol. Arthrosc..

[116] Ra H.J., Ha J.K., Jang S.H., Lee D.W., Kim J.G. (2013). Arthroscopic Inside-out Repair of Complete Radial Tears of the Meniscus with a Fibrin Clot. Knee Surg. Sports Traumatol. Arthrosc..

[117] Nakayama H., Kanto R., Kambara S., Kurosaka K., Onishi S., Yoshiya S., Yamaguchi M. (2017). Clinical Outcome of Meniscus Repair for Isolated Meniscus Tear in Athletes. Asia-Pac. J. Sports Med. Arthrosc. Rehabil. Technol..

[118] Nakanishi Y., Matsushita T., Nagai K., Araki D., Hoshino Y., Kuroda R. (2023). Fibrin Clot and Leukocyte-Rich Platelet-Rich Fibrin Show Similar Release Kinetics and Amount of Growth Factors: A Pilot Study. J. Orthop Surg. Res..

[119] Choukroun J., Diss A., Simonpieri A., Girard M.-O., Schoeffler C., Dohan S.L., Dohan A.J.J., Mouhyi J., Dohan D.M. (2006). Platelet-Rich Fibrin (PRF): A Second-Generation Platelet Concentrate. Part V: Histologic Evaluations of PRF Effects on Bone Allograft Maturation in Sinus Lift. Oral Surg. Oral Med. Oral Pathol. Oral Radiol. Endodontology.

[120] Narayanaswamy R., Sha I. (2022). Arthroscopic Meniscal Repair with Second-Generation Platelet-Rich Fibrin Clot Augmentation. Arthrosc. Tech..

[121] Miron R.J., Zhang Y. (2018). Autologous Liquid Platelet Rich Fibrin: A Novel Drug Delivery System. Acta Biomater..

[122] Elbehwashy M.T., Hosny M.M., Elfana A., Nawar A., Fawzy El-Sayed K. (2021). Clinical and Radiographic Effects of Ascorbic Acid-Augmented Platelet-Rich Fibrin versus Platelet-Rich Fibrin Alone in Intra-Osseous Defects of Stage-III Periodontitis Patients: A Randomized Controlled Clinical Trial. Clin. Oral Invest..

[123] Wu C., Lee S., Tsai C., Lu K., Zhao J., Chang Y. (2012). Platelet-rich Fibrin Increases Cell Attachment, Proliferation and Collagen-related Protein Expression of Human Osteoblasts. Aust. Dent. J..

[124] Sharma R., Sharma P., Sharma S.D., Chhabra N., Gupta A., Shukla D. (2021). Platelet-Rich Fibrin as an Aid to Soft- and Hard-Tissue Healing. J. Maxillofac. Oral Surg..

[125] Miron R.J., Chai J., Zheng S., Feng M., Sculean A., Zhang Y. (2019). A Novel Method for Evaluating and Quantifying Cell Types in Platelet Rich Fibrin and an Introduction to Horizontal Centrifugation. J Biomed. Mater. Res.

[126] Desai T., Babu S.S., Lal J.V., Kaushik Y.S., Lukose A.M., Sandesh G.M., Amaravathi R.S. (2021). Fibrin Clot Augmented Repair of Longitudinal Tear of Medial Meniscus. Arthrosc. Tech..

[127] Borie E., Oliví D.G., Orsi I.A., Garlet K., Weber B., Beltrán V., Fuentes R. (2015). Platelet-Rich Fibrin Application in Dentistry: A Literature Review. Int. J. Clin. Exp. Med..

[128] Vadalà G., Di Giacomo G., Ambrosio L., Cannata F., Cicione C., Papalia R., Denaro V. (2020). Irisin recovers osteoarthritic chondrocytes in vitro. Cells.

[129] Di Giacomo G., Vadalà G., Ambrosio L., Cicione C., Tilotta V., Cannata F., Russo F., Papalia R., Denaro V. (2023). Irisin inhibits tenocytes response to inflammation in vitro: New insights into tendon-muscle cross-talk. J. Orthop. Res..

[130] Vadalà G., Di Giacomo G., Ambrosio L., Cicione C., Tilotta V., Russo F., Papalia R., Denaro V. (2023). The Effect of Irisin on Human Nucleus Pulposus Cells: New Insights into the Biological Crosstalk between the Muscle and Intervertebral Disc. Spine.

